# GPER Signaling in Spermatogenesis and Testicular Tumors

**DOI:** 10.3389/fendo.2014.00030

**Published:** 2014-03-06

**Authors:** Adele Chimento, Rosa Sirianni, Ivan Casaburi, Vincenzo Pezzi

**Affiliations:** ^1^Laboratory of Applied Biology, Department of Pharmacy, Health and Nutrition Sciences, University of Calabria, Cosenza, Italy

**Keywords:** GPER, estrogen receptors, spermatogenesis, germ cells, testicular tumors

## Abstract

Estrogens play important roles in the regulation of testis development and spermatogenesis. Moreover, several evidences suggest that estrogen signaling can be involved in testicular tumorigenesis. The physiological effects of estrogen are mediated by the classical nuclear estrogen receptors ESR1 and 2, which regulate both genomic and rapid signaling events. In the recent years, a member of the seven-transmembrane G protein-coupled receptor family, GPR30 (GPER), has been identified to promote estrogen action in target cells including testicular cells. Ours and other studies reported that GPER is expressed in normal germ cells (spermatogonia, spermatocytes, spermatids), somatic cells (Sertoli and Leydig cells), and it is also involved in mediating estrogen action during spermatogenesis and testis development. In addition, GPER seems to be involved in modulating estrogen-dependent testicular cancer cell growth. However, in this context, the effects of GPER stimulation on cell survival and proliferation appear to be cell type specific. This review summarizes the current knowledge on the functions regulated by estrogens and mediated by GPER in normal and tumor testicular cells.

## Introduction

The three main endogenous estrogens within the testis are 17β-estradiol (E2), estrone (E1), and estriol (E3) among which the predominant and most active steroid is E2. This steroid is mainly obtained by the conversion of testosterone through the activity of the enzyme complex named aromatase cytochrome P450C19 A1, encoded by the *CYP19* gene (1) whose expression is under the control of different tissue-specific promoters (2). In the testis, aromatase expression is transcriptionally regulated by the interaction of different transcription factors (3–10) to specific functional motifs identified within the P.II promoter region (2).

Physiological effects of estrogens are mediated by the classical nuclear estrogen receptor alpha (ESR1) and estrogen receptor beta (ESR2), which mediate both genomic and rapid signaling events (11). In addition, estrogens induce rapid non-genomic responses through a membrane-associated G protein-coupled receptor also named GPR30/GPER that has been identified as a novel estrogen receptor (ER) (12). Several studies performed on aromatase-deficient patients (13) and on aromatase or ERs knocked-out mouse models (14) have confirmed that estrogens play key roles in the development and maintenance of normal reproductive function and fertility as well as in pathological processes (15–18). Moreover, aromatase overexpression in mice leads to infertility in either all male or in 50% of them when it takes place in fetal life or at puberty, respectively (19). Therefore, a delicate balance between androgens and estrogens, partially controlled by aromatase activity, seems to be essential for the maintenance and control of normal spermatogenesis (15, 20).

Spermatogenesis is a complex process under the control of gonadotropins luteinizing hormone (LH) and follicle-stimulating hormone (FSH) as well as testosterone and different locally produced factors (21) including estrogens (15, 17, 22). It is now accepted that E2 regulates all the events related to spermatogenesis including gonocyte and spermatogonia proliferation, meiosis, Sertoli cell function as well as spermiation, sperm transport, and epididymal sperm maturation (23).

Noteworthy, altered hormonal status has been associated with initial malignant transformation of germ cells (24–26). Accordingly, a relationship between testicular germ cell cancer (TGCC) and maternal estrogen/androgen levels in early pregnancy has been documented (27). It has been hypothesized that early arrest of gonocyte differentiation followed by an increase in cell proliferation could determine genomic aberrations (28) responsible for transformed pre-carcinoma *in situ* (CIS), also known as intratubular germ cell neoplasia unclassified (29). Recently, an association of polymorphic variants in genes encoding for ESR1, ESR2, and LH receptors with TGCC risk and metastasis has been demonstrated (30). In addition, an elevated GPER protein expression was revealed in all intratubular germ cell tumors, seminomas, and embryonal carcinomas (31) as well as in testicular stromal neoplasms (32–34). However, the molecular mechanisms involved in the initiation and progression of testicular cancers are still under investigation.

This review will focus on the roles of estrogenic signaling in spermatogenesis and testicular tumors, with special emphasis on rapid mechanisms of action mediated by the novel ER GPER.

## Estrogens Receptors and GPER Expression in the Testis

Testicular estrogens exert their functional role through the interaction with estrogen receptors ESR1 and ESR2, encoded by two different genes located in humans on chromosomes 6 and 14, respectively (11).

Within the testis, ESR1 and ESR2 expression is highly variable, with major differences between species, as well as between individuals within a species (17). Studies on the immunohistochemical localization and mRNA expression of the receptors in testicular tissues and cells reported divergent data (35–37). The reasons for these discrepancies could be attributed primarily to tissue preservation techniques and/or to antibodies used for immunohistochemical analysis (38, 39).

In the mouse testis, ESR1 was found only in Leydig cells and in some peritubular myoid cells, whereas ESR2 was revealed in Leydig cells, Sertoli cells, and germ cells, particularly spermatocytes (40, 41). Generally, while ESR1 expression was recovered in the interstitial space, ESR2 has been observed within the seminiferous epithelium. However, Lucas and coworkers (39) have confirmed ESR1 expression also in Sertoli cells.

At first, ESR1 immunodetection in rats was restricted to Leydig cells (42) but later its expression was reported in the seminiferous compartment (43), in the immature Sertoli cells (39, 44), in whole adult testis, and in purified germ cells (45). Regarding ESR2, there is a general consensus on its localization in the seminiferous tubules but there are conflicting data regarding its presence in germ cells (17, 46). Indeed, more recently, ESR2 expression in rat pachytene spermatocytes (PS) (45) and spermatids was revealed (47).

The presence of ERs in human testicular cells is well-documented (48, 49). It was speculated that the most susceptible cells to the actions exerted by estrogenic ligands are round spermatids (RS), where ESR2 content levels are the higher (50). In particular, in men the full-length protein ESR1 (66 kDa) and one isoform lacking exon 1 (46 kDa) have been identified in isolated immature germ cells (49). For ESR2, two proteins which correspond to the long (60 kDa) and short (50 kDa) forms have been detected in germ cells (48). The presence of ESR1 and ESR2 has also been reported in the human ejaculated spermatozoa (49, 51).

GPER has been identified in a variety of human and rodent estrogen target tissues (52–56). Studies related to GPER intracellular localization revealed its presence in the endoplasmic reticulum, Golgi apparatus (54), plasma membrane (57), and nuclei (58, 59). Using a *Gper*-lacZ reporter mouse, Isensee et al. (60) demonstrated extensive expression of GPER in several endocrine organs including the testis. This agrees with our studies that revealed GPER expression in a mouse spermatogonia cell line (GC-1 cells) (61), in adult rat PS (45) and in rat RS (47) suggesting a role for this receptor in spermatogenesis (62). Moreover, GPER expression has also been recently demonstrated in Sertoli cells (63, 64). In mice, it has been claimed that GPER is not involved in estrogenic responses of the reproductive organs (65). Indeed, these authors generated GPER-deficient (GPERKO) mice and showed that mutant male and female are fertile. However, it is noteworthy that data on the spermatogenetic process are missing and a careful examination of estrogenic response was carried out only in the uterus and mammary gland. It should be noted, however, that GPR30 plasma membrane-association and activation by E2 to invoke intracellular signaling or cell proliferation have not been demonstrable by some laboratories, and that the subcellular localization of the receptor and the identity of its biologically important ligands continue to be the subject of considerable debate (66–69).

## Estrogen Receptor Signaling and Mechanisms of Action: Role of GPER

Classically, once activated ERs act as transcription factors to modulate the expression of target genes through the interaction with estrogen response elements (EREs) within their promoter region. However, ERs can also regulate gene expression without directly binding to DNA, through protein–protein interactions with other transcription factors in the nucleus (70). In addition, membrane-associated ERs mediate non-genomic actions of estrogens, which can lead to regulation of gene expression through the activation of kinases signal-transduction pathways that eventually act on target transcription factors (70). Moreover, ERs can be targets of mitogen-activated protein kinase (MAPK) signaling pathway (71) indicating that non-genomic pathways activated by estrogens can modulate the functions of ERs themselves (70).

It is currently known that estrogen non-genomic actions can be mediated by GPER, in a wide number of normal and neoplastic cells (12, 72). Following the first reports in 1997 on its identification (73–75), it has been subsequently demonstrated that estrogen through GPER rapidly activates different pathways including EGFR transactivation leading to the rapid phosphorylation of the MAPKs ERK1/2 (76–78), stimulation of adenylyl cyclase (52, 55), mobilization of intracellular calcium (Ca^2+^) stores, and phosphoinositide 3-kinase (PI3K) signaling pathways activation (54). Regarding the G proteins involved in GPER-mediated signaling, an important role for Gαs (55) and Gβγ (76) has been suggested. Gαs was shown to be responsible for adenylate cyclase stimulation and consequently cAMP increase (55), while Gβγ subunit and the downstream Src-related tyrosine kinases activation were involved in MAPK transduction pathway (76). Thus, through these rapid pathways, GPER modulates transcription of different genes such as c-fos, connective tissue growth factor (CTGF), and early growth response protein 1 (Egr1) (53, 79–81).

## ERs and GPER Non-Genomic Signaling in Testicular Cells: Role in Spermatogenesis Regulation

The important role of estrogens in the regulation of spermatogenesis was evidenced by *in vivo* studies performed with knock-out (KO) mouse models for the estrogen receptors as well as for the aromatase gene.

Esr1 KO (αERKO) animals have reduced fertility because of abnormal fluid reabsorption in the efferent ductules (82), whereas in Esr2 KO (βERKO) animals (14), spermatogenesis, steroidogenesis, and fertility were initially found unaffected. Indeed, the Esr2 null mice displayed alternative splicing transcripts that could functionally compensate for the lack of full-length receptors. An Esr2 null mouse, lacking any ERβ isoform, was generated by Cre/LoxP-mediated excision of Esr2 exon 3 (83). Although the causes are still unknown, these mice are infertile despite the morphofunctional characteristics of their gonads and spermatozoa appearing normal.

The absence of estrogen production observed in the aromatase knock-out (ArKO) mice, causes a more severe testicular phenotype compared to both ERKO mice, with a decreased number of spermatocytes and round and elongated spermatids (84, 85). Data from ArKO mice support the hypothesis that an alternative receptor (i.e., GPER) and alternative pathways could be involved in mediating estrogen effects on spermatogenesis. However, GPER was not considered to be involved in estrogenic responses of reproductive organs since GPERKO male as well as female mice were found fertile (65). A careful analysis of the study by Otto et al. will show that data on the spermatogenetic process are missing, and an examination of the estrogenic response was carried out only on uterus and mammary gland. Thus, the generation of a triple KO (ESRs and GPER) would be useful to highlight, or eventually to flush out, the cross-talk and functional redundancy between the three different receptors as well as between genomic and non-genomic effects exerted by estrogen in the modulation of spermatogenesis. Understanding this difference could be very important especially given that the loss of non-genomic ESR1 signaling pathway is responsible for most of the reproductive tract defects observed in αERKO mice (86). These data support the hypothesis that rapid estrogen signaling plays a crucial role in spermatogenesis.

In this regard, Chieffi et al. demonstrated, in non-mammalian vertebrate models, frog *Rana esculenta* and *Podarcis s. sicula* the involvement of ERK1/2 signaling in spermatogonial cell proliferation following E2 treatment (87, 88). Accordingly, studies with the mouse spermatogonial GC-1 cell line showed that estradiol rapidly activates a proliferative pathway involving EGFR/ERK/fos/cyclin D1 requiring a functional cross-talk between GPER and ESR1 (61). In fact, c-*fos* up-regulation and ERK1/2 activation by estradiol or by selective agonists for ESR1 (PPT) or GPER (G-1) were overcome by the presence of the pure ESR1 antagonist ICI 182780, or by GPER gene silencing (61).

In rat PS, estrogens can directly activate rapid signaling pathways controlling spermatogenesis. Specifically, our studies performed in rat primary cultures of PS demonstrated that estradiol, working through both ESR1 and GPER, activates the rapid EGFR/ERK/c-jun signaling cascade, which in turn triggers the mitochondrial apoptotic pathway concomitantly with an increased expression of bax and a reduction of cyclin A1 and B1 protein levels (45). Similarly, in a mouse PS-derived cell line (GC-2 cells) we showed: (i) an expression pattern for ESR1, ESR2, and GPER similar to primary rat PS (89), (ii) ESR1- and GPER-dependent cell growth inhibition, and (iii) a reduction in cyclin D1 and B1 protein expression and an increase in p21 protein content. As in primary PS, these events anticipated an apoptotic mechanism that was studied in detail. It required the activation of MAPK family members ERK1/2, JNK, and p38, followed by activation of intrinsic apoptotic pathway determining bax up-regulation, cytochrome c release, caspase 9 and 3 activation, parp-1 cleavage, and DNA fragmentation.

Estrogen receptors and GPER non-genomic signaling were investigated also in primary cultures of adult rat RS in order to clarify the role of their activation in the maturation and/or apoptosis of these cells. Particularly, in RS, estrogen through a functional cross-talk between GPER and ESR1 is able to activate EGFR/ERK pathway involved in the transcriptional modulation of genes controlling apoptosis and differentiation such as cyclin B1 and bax (47).

Moreover, non-genomic effects of estrogens have been also evaluated in rat immature Sertoli cells. In this cell type, treatment with estrogen induced a rapid translocation of ESR1 and ESR2 to the plasma membrane, together with the activation of proto-oncogene tyrosine kinase Src, which in turn phosphorylates and activates EGFR/MAPK3/1 pathway responsible for the enhanced cyclin D1 (CCND1) gene transcription and cell proliferation (39). Once activated, the GPER/EGFR/MAPK3/1 signaling cascade caused an increase in Bcl-2 protein content and a decrease in bax expression, suggesting that in immature Sertoli cells, the anti-apoptotic effects of E2 are mediated by GPER activation (63, 64). More recently, Royer and coworkers (90) clarified ESRs and GPER downstream pathways involved in rat immature Sertoli cells’ proliferation and apoptosis. Specifically, these authors showed that ESR1 activated by its ligand rapidly induces EGFR/ERK1/2 and PI3K pathways that in turn increase cyclin D1 expression responsible for Sertoli cell proliferation (90). Downstream of GPER, after E2 or G-1 treatment, they showed activation of EGFR/ERK1/2/phopho-CREB and PI3K pathways leading to anti-apoptotic effects by upregulating BCL-2 and BCL-2L2 proteins and decreasing bax expression (90).

In summary, these studies suggest that estrogen can influence, in a cell-specific manner, all the biological features that characterize the spermatogenetic process such as germ cell proliferation, differentiation, as well as germ cells survival and apoptosis. In addition, another interesting aspect is that genomic and rapid pathway can work independently but cooperate to reach the same goal as evidenced in Sertoli cells where E2-genomic action on cyclin D1 induces proliferation while E2 rapid action through GPER activates anti-apoptotic signals (90).

## Role of Estrogen Receptors in Testicular Cancer

Estrogen receptors play important roles in the modulation of several types of tumors, such as those of the breast, endometrium, ovary, adrenal, prostate, colon, liver, lung as well as testis.

### ERs and germ cell cancers

Among all the malignant tumors of the testis, 95% are type II germ cells cancers (TGCC), which are classified into two sub categories seminoma and non-seminoma, both derived from a common precursor cell called CIS (91).

Recently, it has been reported that polymorphisms in ESR1, ESR2, and luteinizing-hormone-releasing hormone (LHRH) genes were linked to TGCC risk and metastasis occurrence. In particular, two *ESR2* and *LHRH* genetic variants were related to TGCC-reduced risk, while one polymorphism in *ESR1* or *LHCGR* (LH/choriogonadotropin receptor) gene was associated with an increased risk for TGCC (30). It is known that *LHRH* is primarily expressed in human fetal Leydig cells (92) and that its actions are important for postnatal Leydig cell differentiation (21). Moreover, Leydig cell function and spermatogenesis is impaired in men with CIS of the testis (93). In addition, it has been well-documented that ESR2 is expressed in Sertoli cells, Leydig cells, and gonocytes (15, 94) and that exposure to elevated estrogen levels can affect germ cells either directly or indirectly through adverse effects on both Sertoli cells and Leydig cells (95). Thus, polymorphisms in genes encoding *ESR2* and *LHRH* may influence the sensitivity of these cells to estradiol- and LH-mediated effects and influence TGCC development.

It is known that elevated pituitary and steroids hormone levels play an important role in the malignant transformation of pre-CIS into seminomatous (SE) and non-seminomatous (NSE) neoplasms. Indeed, patients with severe hypogonadotropic hypogonadism do not show TGCC and in patients with complete androgen insensitivity, gonocytes remain undifferentiated, similar to that seen in CIS (96–98). Noteworthy, a polymorphism in ESR1 identified by Brokken and coworkers (30) is associated with higher levels of LH in healthy control subjects, indicating that levels of gonadotropins influence the progression of CIS to either SE or NSE. However, despite the observation that these subjects had higher testosterone levels, there was not a statistically significant association between androgen levels and CIS progression (30).

In the attempt to define a role for GPER in TGCC, Franco and coworkers (99) have evaluated its expression in post-puberal TGCCs (30 seminomas, 5 teratomas, 12 embryonal carcinomas, and 20 intratubular germ cell tumors) by immunohistochemical analysis. GPER protein expression seemed to be high in all intratubular germ cell tumors, seminomas, and embryonal carcinomas, whereas in teratomas, the immunoreactivity was low. Western blot analysis, performed on the same category of samples, showed a good correlation with the immunohistochemical data (32). GPER protein expression and activation was also evaluated in estrogen receptor alpha-66 negative TCam-2 (100) and JKT-1 (101) human seminoma cell lines, both isolated from type II TGCC. Recently, Wallacides and coworkers (102) have demonstrated that in TCam-2 cells, both estradiol and testosterone (after conversion to E2) can stimulate cell proliferation in the absence of ESR1. The pathway involved is GPER/protein kinase A (PKA)/CREB, which enhanced expression of estrogen receptor alpha-36, a truncated isoform of the canonical ESR1 that in turn is necessary for both EGFR membrane localization and E2-mediated stimulation of EGFR expression (102). These results agree with those reported by Zhang and coworkers (103) showing estrogen receptor alpha-36 as mediator of E2-dependent signaling in ER-negative breast cancer cells. Then, tumors that lack ESR1 66 can still retain an estrogen mitogenic signaling. In addition, breast tumors treated with tamoxifen to block the classical ESR1 signaling often acquire resistance to this drug. These resistant tumors show increased activity of both ESR1 36 and GPER (104).

Conversely, in JKT-1 cells, estradiol inhibits cell proliferation through an ESR2-dependent mechanism that is completely suppressed by the ER antagonist ICI182780 (105). However, JKT-1 cells also express GPER and treatment of these cells with G-1, which has low affinity for ESR2 (106), induced JKT-1 cell proliferation (107, 108). It has been reported in some models that GPER and ERs (ESR1/ESR2) or truncated splice variant of ERs could either cooperate or cross-talk (109, 110). ESR1 is not expressed in JKT-1 cells (105) and ESR2 is not localized at the extracellular membrane as shown by western blot analysis after subcellular fractioning (107). In addition, using RNAi silencing and G15, a selective GPER antagonist (111), the involvement of GPER in xenoestrogen-, bisphenol A-, and E2 coupled to bovine serum albumin (E2-BSA)-induced JKT-1 cell proliferation was definitively demonstrated (112, 113). The clear difference between E2-mediated proliferation in TCam-2 cells and E2-dependent growth inhibition in JKT-1 cells could be explained by different expression levels of GPER and estrogen receptor alpha-36, or by a different expression pattern of ER cofactors between the two cell lines. In fact, it has been previously demonstrated that JKT-1 cells lack expression for most of the genes detectable in type II TGCC (114, 115), thus providing evidences for great differences between TCam-2 and JKT-1 cells.

### GPER, xenoestrogens, and germ cell cancer

Xenoestrogens are part of the endocrine-disrupting chemicals (EDCs), hormone-like compounds widespread in the environment that mimicking the natural hormone estradiol interfere with endogenous endocrine regulation at specific stages, such as during fetal growth causing hypofertility and/or testicular germ cancer. Most of EDCs have a very weak affinity for binding to ERs (116–118), though, these compounds mediate endocrine disruption in both animals and humans at low environmental concentrations (119, 120).

It has been reported that EDCs act through hormone-independent mechanisms (121) or through a non-genomic membrane-initiated signaling pathway activated via membrane-localized ERs (122–126). It has been demonstrated that pesticides considered as estrogenic EDCs are able to activate GPER signaling (113, 127). In particular, bisphenol A (BPA), an organochloride pesticide, via GPER is able to stimulate JKT-1 cell proliferation, through a rapid activation of cAMP-dependent PKA and cGMP-dependent protein kinase G (PKG) signaling pathways associated with phosphorylation of the transcription factor CREB and the cell cycle regulator pRb (113).

Furthermore, several reports also suggested that BPA binds to GPER and mediates rapid estrogenic actions (52, 54, 106). Recently, in JKT-1 cells, Chevalier and coworkers (112) showed that treatment with G-1, BPA, and very low doses (nanomolar) of E2-BSA, determined an increase in seminoma cell growth through a non-genomic GPER-dependent mechanism involving PKA and MAPK pathways. Opposite effects were produced by E2 that at physiological concentrations, binding intracellular ESR2 through a classical genomic mechanism, suppressed *in vitro* JKT-1 cell proliferation (112). In addition, E2-BSA and BPA-mediated proliferative effects were not neutralized by ICI182780. The presence of G15 instead abrogated E2-BSA and BPA-mediated effects on seminoma cell growth, confirming that estrogens and xenoestrogens through classical ERs and GPER can activate distinct genomic and non-genomic pathways depending on their relative affinity for the receptors and on cofactor expression within the cells. However, other authors (128) demonstrated that high doses of BPA upregulate expression of Fas and FasL, and active caspase-3 in the mouse testis, determining apoptosis of Leydig cells and germ cells. It is evident that BPA effects depend on the dose. At low concentration (10^−9^ M), BPA allows the non-genomic effect to be displayed because of the high affinity of BPA for GPER. At high micromolar concentrations, BPA may trigger a suppressive effect via ESR2, which neutralizes the non-genomic effect. When mixed together at low concentrations, BPA and E2 are mutually antagonistic.

Collectively, these data confirm that EDCs through a GPER-mediated non-genomic mechanism are involved in testicular germ cell carcinogenesis and that their impact in this context may depend on (i) the relative expression of receptors (ERs and GPER), (ii) the endogenous concentration of E2, and (iii) the relative binding affinities to ERs or GPER.

### GPER and testicular stromal neoplasms

Several studies reported GPER expression in testicular stromal neoplasms such as Leydig and Sertoli cell tumors (32–34). Sertoli cell tumor is a rare type of sex cord-gonadal stromal tumor accounting for no more than about 1% of all testicular tumors. Sertoli tumors are characterized by hyperestrogenism due to a direct production and/or conversion of testosterone to estrogen by the tumor (129). However, a possible coordinated regulation or a cross-talk between ERs and/or GPER was not investigated on neoplasmic samples at different stages of the disease. A previous study has evidenced a differential expression pattern of the classical ERs in human normal and neoplastic Leydig cells with the exclusive presence of ESR1 in tumor cells, which could amplify estrogen signaling and could contribute to tumor growth (9, 34). In fact, we have previously shown that Leydig tumors produce estrogens that bind to ESR1 and activation of this receptor sustains cell proliferation (9). We also have shown that ERs antagonists such as hydroxytamoxifen (OHT) and ICI182780 are able to reduce proliferation of a rat Leydig tumor cell line. Similar effects were also found using letrozole, an aromatase inhibitor. However, treatment of cancers with anti-estrogens frequently causes drug resistance (130). Indeed, a new treatment for Leydig cell tumors is deemed.

In a recent paper, we have showed that GPER is a good target to reduce Leydig tumor proliferation. In fact, GPER is expressed in this type of cancer and its activation is associated with a drastic reduction of cell proliferation (131). In particular, using R2C, a rat tumor Leydig cell line, we have demonstrated that GPER activation by G-1 is associated with the initiation of the intrinsic apoptotic mechanism. Apoptosis after G-1 treatment was evidenced by the appearance of DNA condensation and fragmentation, decrease in Bcl-2 and increase in Bax expression, cytochrome c release, and caspase and PARP-1 activation. These effects were dependent on GPER activation since silencing of the gene, using a specific siRNA, prevented cytochrome c release, Bax increase, Bcl-2 decrease, PARP-1 activation, and decrease in cell proliferation. These events required a rapid however sustained ERK1/2 activation (131). Our data are consistent with previous reports demonstrating that transient activation of ERK1/2 plays a pivotal role in cell proliferation and that sustained ERK1/2 activation induces cell cycle arrest (132) and death (133–135). The ability of G-1 to reduce the growth of R2C *in vitro* was also evaluated *in vivo*. G-1 significantly inhibited the growth of R2C xenografts and increased the number of apoptotic cells. To address if the use of G-1 for the therapy of Leydig tumors could indeed affect normal spermatogenesis, we evaluated G-1 effects on testis morphology. Our *in vivo* experiments demonstrated that administration of G-1 for more than a 2-week period did not cause any damage to the normal testis structure, opposite to that seen with Cisplatin. GPER activation induces proliferation of spermatogonia (61), which represent the stem cells of male germ cells. It could then be speculated that the use of a GPER-specific agonist for the therapy of Leydig tumors would not affect normal spermatogenesis allowing preservation of fertility in patients treated for this type of tumor. On the other hand, chemotherapeutic agents currently used for the treatment of testicular cancers, such as Cisplatin, despite their potent anti-neoplastic action, have several side effects including nephrotoxicity (136), peripheral neuropathy (137), and azoospermia (138). This last event is dependent on a reduction in the number of spermatogonia, which appear to be the most sensitive germ cell type to cisplatin (139).

Although further studies are needed, our results point out how GPER and its agonists such as G-1 can be considered as a potential new pharmacological tool to reduce the growth of Leydig cell tumors. This drug, opposite to those currently used, does not seem to affect germ cells and thus could preserve male fertility.

## Concluding Remarks

**Figure 1:**
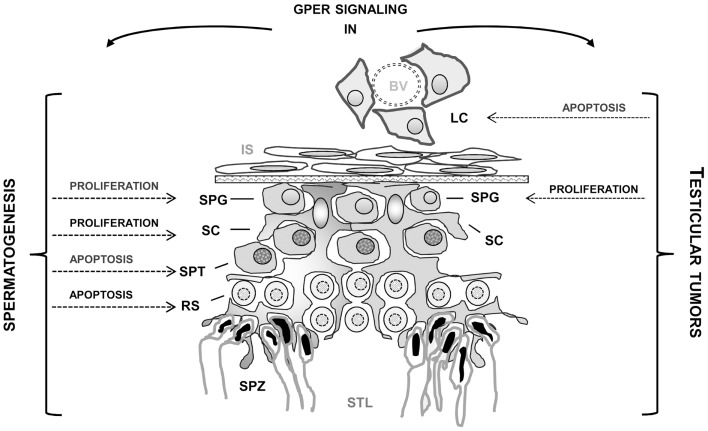
**Effects mediated by GPER activation in normal and tumoral testicular cells**. BV, blood vessel; LC, Leydig cells; IS, interstitial space; SC, Sertoli cells; SPG, spermatogonia; SPT, spermatocytes; RS, round spermatids; SPZ, spermatozoa; STL, seminiferous tubule lumen.

Several studies carried out in the past years showed that in the testis, a regulated balance between androgens and estrogens seems to be essential for normal testicular physiology and reproduction. Another important finding is the widespread presence of ESR1 and ESR2 in both somatic and germ testicular cells. In addition, the discovery of the new transmembrane estrogen-binding protein GPER in the testis has opened new perspectives to better understand the rapid membrane pathways induced by estrogens. In fact, estrogenic activity appears to involve not only the classical genomic pathway, but also rapid membrane receptor-initiated pathways and possibly non-classical nuclear ER-tethering pathways. Estrogen actions on spermatogenesis appear to influence, in a cell-specific manner, germ cell proliferation, differentiation, as well as germ cell survival and apoptosis (Figure 1). Another interesting aspect revealed by very recent studies is that genomic and rapid pathways can work independently but cooperate to reach the same goal. An intriguing observation is that in testicular cells both rapid and genomic mechanisms via nuclear and membrane-associated ERs can be differentially triggered by xenoestrogens based on their concentration. Indeed, to further investigate the precise impact of those chemicals, alone or more importantly in a mixture, the development of human *in vitro* testicular models is required.

In addition, the recent immunolocalization of GPER in testicular tumors and the reports indicating that GPER activation by selective ligands can allow for opposite outcomes in different testicular cells (i.e., seminoma and Leydig cells) (Figure 1) should open new perspectives to define the mechanisms behind the development of estrogen-dependent testicular tumorigenesis as well as to provide a new target for the development of new pharmacological tools against testicular cancer.

## Conflict of Interest Statement

The authors declare that the research was conducted in the absence of any commercial or financial relationships that could be construed as a potential conflict of interest.
